# In Situ Reconstructed Crystalline–Amorphous CuNi Nanotubes Unifying Activity and Stability for Oxygen Evolution

**DOI:** 10.1002/advs.76436

**Published:** 2026-07-06

**Authors:** Shi‐Yu Zhu, Han Gao, Meng Li, Ling‐Rui Wang, Tian‐Yu Xia, Wei‐Di Liu, Xiao‐Lei Shi, Hai‐Zhong Guo, Zhi‐Gang Chen

**Affiliations:** ^1^ School of Physics Zhengzhou University Zhengzhou China; ^2^ School of Chemistry and Physics ARC Research Hub in Zero‐emission Power Generation for Carbon Neutrality and Centre for Materials Science Queensland University of Technology Brisbane Queensland Australia; ^3^ Institute of Quantum Materials and Physics Henan Academy of Sciences Zhengzhou China

**Keywords:** crystalline–amorphous heterostructure, CuNi nanotubes, in situ reconstruction, noble‐metal‐free electrocatalysts, oxygen evolution reaction

## Abstract

Development of durable and active noble‐metal‐free catalysts for the alkaline oxygen evolution reaction (OER) remains a critical challenge. Conventional electrocatalysts typically suffer from an intrinsic trade‐off between activity and stability. Here, we report an in situ electrochemically reconstructed CuNi nanotube with a crystalline–amorphous heterostructure, featuring a conductive crystalline alloy core encapsulated by an amorphous nickel oxide shell. The metallic core provides a high‐conductivity framework for rapid charge transfer and maintains structural integrity, while the in situ derived amorphous phase maximizes the density of accessible active sites and passivates the metallic core against oxidative corrosion. The optimized catalyst achieves an overpotential of 229 mV at 10 mA cm^−2^ and operates stably for over 1000 h in alkaline OER, reconciling the catalytic activity and stability. Mechanistically, operando Raman spectroscopy and electron energy loss spectroscopy elucidate the dynamic valence modulation and surface phase evolution as the origin of its performance. Overall, this work establishes controlled electrochemical reconstruction as a transformative paradigm for unifying catalytic activity and stability, providing a design principle for advanced, earth‐abundant OER electrocatalysts.

## Graphical abstract

Controlled electrochemical reconstruction creates a crystalline–amorphous CuNi nanotube that unifies catalytic activity and durability for alkaline oxygen evolution. The conductive metallic core and active amorphous oxide shell work synergistically to deliver low overpotential and exceptional stability exceeding 1000 h, offering a new strategy for earth‐abundant OER catalysts.

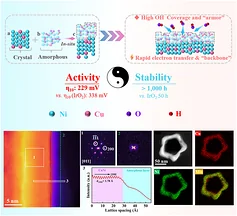

## 1 Introduction

The sluggish kinetics of the oxygen evolution reaction (OER) fundamentally limit the efficiency of water electrolysis for green hydrogen production. Although noble‐metal catalysts deliver high OER efficiency performance, their resource scarcity limits practical applications, prompting a shift toward earth‐abundant metals (e.g., Ni, Fe, Co) [1, 2, 3]. Nevertheless, these noble‐metal‐free catalysts typically suffer from surface over‐oxidation and subsequent dissolution under harsh alkaline conditions, resulting in structural degradation and poor stability [4]. To address this challenge, recent efforts have focused on comprehensive strategies to improve both catalytic activity and stability, including tailoring material composition (e.g., metal sites [5, 6], oxide sites [7], nitride/sulfide sites [8, 9]), microstructures (e.g., lattice defects [10], geometric sites [11], coordinatively unsaturated sites [12]), and electronic properties (e.g., doping [13], metal‐support interactions [14], heterointerfaces [15, 16]).

Heterostructure engineering has emerged as an effective strategy to simultaneously enhance catalytic activity and stability by integrating distinct components and constructing functional interfaces [17]. Particularly, crystalline–amorphous heterostructures have gained intensive attention because they synergistically leverage the high conductivity and structural integrity of crystalline components together with the defect‐rich, high‐density active sites of amorphous domains [18, 19]. Crystalline materials, such as alloys, ensure efficient electron transfer and mechanical resilience, while amorphous layers, such as Ni/Co oxides, facilitate adsorption of oxygen intermediates [20, 21]. Moreover, their inherent self‐adaptive behavior under electrochemical conditions suppresses structural degradation [15, 22, 23, 24]. This leads to dramatically enhanced stability, which holds significant implications for the long‐term operation of noble‐metal‐free catalysts. Recently, Lu et al. broke the activity–stability trade‐off in OER catalysts by constructing a heterostructure comprising an outer multicomponent amorphous oxide layer and an inner self‐replenishing layer, which enables stable operation for over 1600 h at an industrial‐level current density [25].

Compared with stepwise ex situ assembly, in situ construction of crystalline–amorphous heterostructures provides superior crystallographic coherence and active interfacial electronic coupling [26, 27]. Recent advances have demonstrated that surface reconstruction induced by ligand exchange or external stimuli, such as electric fields, can generate functional interfaces/surfaces during catalysis [28, 29]. For example, Gao et al. reported the one‐step self‐assembly of Co‐W oxide nanostructures on copper oxide substrates. The resulting catalyst exhibits pronounced dynamic self‐optimization behavior, thereby significantly enhancing OER performance in alkaline media [30]. As a rising approach, in situ reconstruction requires precisely tailored templates (precursor structures) and suitable activation protocols, making it scientifically significant while methodologically challenging. Recently, Sun et al. successfully transformed polycrystalline Cu_2_O nanoparticles into pure copper catalysts with distorted nanotwins via electroreduction, achieving hydrogen evolution reaction performance surpassing commercial Pt/C in acidic electrolytes [31]. Although in situ reconstruction has been well recognized for tailoring new‐class electrocatalysts, comprehensive understanding of the evolution process remains inadequately explored, yet is essential for rational catalyst design.

In this study, we present a potential‐driven in situ reconstruction strategy to fabricate crystalline–amorphous CuNi nanotubes (NTs) as the catalyst reconciling activity and stability in OER. Comprehensive transmission electron microscopy (TEM) and electron energy loss spectroscopy (EELS) characterizations reveal their hollow one‐dimensional features and spatially resolved electronic structure. This unique heterostructure integrates abundant accessible active sites with a protective amorphous shell, enabling a synergy between high activity and long‐term stability. As an earth‐abundant metal catalyst, it delivers an overpotential of 229 mV at 10 mA cm^−2^ and impressive stability in OER, rivaling noble‐metal benchmarks. Operando spectroscopic characterizations further capture the dynamic surface reaction. Density functional theory (DFT) calculations suggest that interfacial coupling in CuNi@NiOOH strengthens intermediate adsorption and accelerates OER kinetics. This work offers a conceptual framework for designing advanced and economical OER electrocatalysts through heterostructure engineering and a reconstruction paradigm.

## 2 Results and Discussion

Figure 1a highlights the conceptual design of crystalline–amorphous heterostructures generated via in situ reconstruction. This architecture integrates rapid electron transport with adaptive surface stability, thereby reconciling catalytic activity and stability. Figure 1b schematically illustrates the potential‐driven reconstruction process with dual features—knockout of OER‐inert Cu atoms in the nanowire (NW) along with surface reconstruction for active Ni species. The NW–NT transformation was ex situ characterized using TEM during the potential cycling process (Figure 1c and Figure S1). Initially, irregular corrosion is observed on the NW surface after 500 cycles of cyclic voltammetry (CV) activation (Figure S1), indicating electrochemical removal of partial Cu atoms. As illustrated in Figure 1c, prolonging to 1000 cycles, the Cu content further decreases while the Ni shell remains well‐preserved. Upon extending the cycling to 1500–2000 cycles, the etched regions acceleratingly expand and ultimately transform the NW into the NT structure. Additional data collected at 1900, 2100, and 2300 cycles confirm that the structure can be considered as the steady state at ∼2000 cycles (Figures S2–S4). The variation trend in compositions during this process is summarized in Figure S5. The atomic knockout process followed nonlinear kinetics, with initial surface defects inducing localized oxidation and, upon formation of porous side channels after ∼1000 cycles, enabling rapid atom migration and accelerated structural reconstruction [32].

**FIGURE 1:**
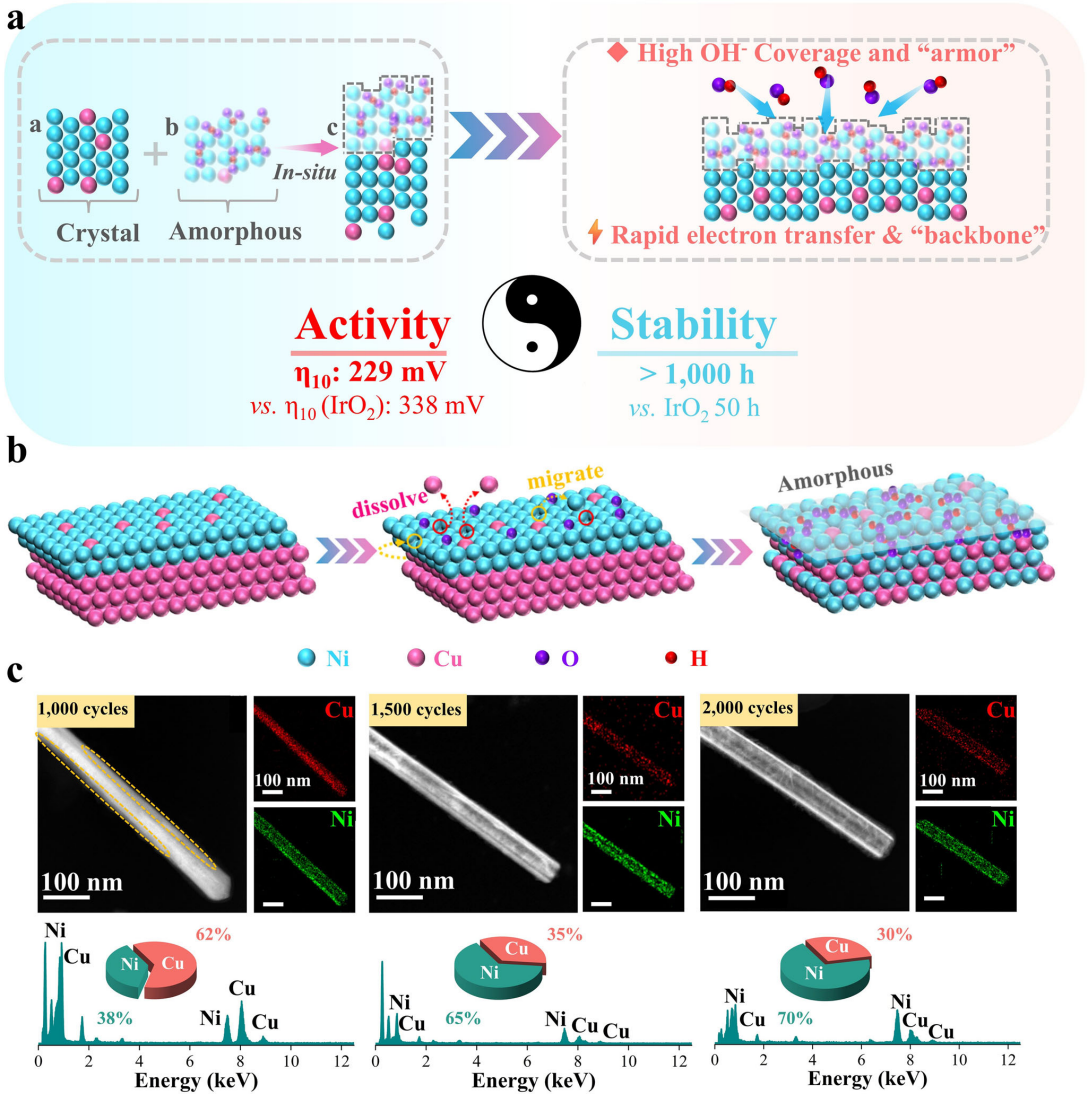
Catalyst design concept highlighting in situ structural reconstruction and OER improvement. (a) Conceptual illustration of crystalline–amorphous heterostructures formed via in situ reconstruction, balancing catalytic activity and stability. (b) Schematic illustrations of the in situ transformation from the CuNi NW to the CuNi NT. (c) Morphological and compositional change in one‐dimensional structures driven by potential cycles.

The precursor NW is crucial for subsequent reconstruction, and necessary TEM characterization has been performed on the CuNi NWs. A low‐magnification TEM image in Figure 2a reveals uniform CuNi NWs with an average diameter of ∼50 nm and micrometer‐scale lengths. In Figure 2b, the high‐angle annular dark‐field scanning transmission electron microscopy (HAADF‐STEM) image and corresponding fast Fourier transform (FFT) pattern confirm a [001] oriented face cubic center (*fcc*) structure with a measured (020) *d*‐spacing of *fcc*‐Cu. Atomic‐scale HAADF‐STEM image in Figure 2c reveals coherent core–shell interfaces with minimal lattice strain, which is analyzed by the geometric phase analysis (GPA) method in Figure S6, supporting epitaxial Ni growth on Cu. Lattice intensity profiles (Figure 2d) along the [110] direction show *d*‐spacings in the two regions, which can be attributed to Cu and Ni, respectively. Energy dispersive x‐ray spectroscopy (EDS) identifies Cu‐rich cores and Ni‐rich shells, with a Cu/Ni ratio of ∼75:25 consistent with inductively coupled plasma mass spectrometry (ICP‐MS) results (Figure 2e,f, Figure S7 and Table S1). In addition, the NWs were ultra‐thin sectioned and observed from the top view, as shown in Figure S8. The quasi‐pentahedral, core‐shell configuration is better clarified, and a five‐fold twin structure can be determined, which is an appealing feature reported in low‐dimensional *fcc* metals [33, 34]. Powder x‐ray diffraction (PXRD) further confirms the *fcc* structure (Figure S9), with a shoulder peak at ∼44.2° supporting the presence of compositionally segregated Cu–Ni phases [35]. The core‐shell configuration arises from sequential reduction kinetics: the higher reduction potential of Cu^2+^/Cu (0.34 V vs. RHE) favors Cu nucleation, followed by co‐reduction of Ni^2+^ and residual Cu^2+^ to form the epitaxial Cu–Ni shell [36]. Furthermore, EELS and x‐ray photoelectron spectroscopy (XPS) probe the valence states and electronic structure across the core–shell interface, as shown in Figures S10 and S11. This well‐defined core–shell architecture provides a robust, strain‐matched template for subsequent potential‐driven reconstruction.

**FIGURE 2:**
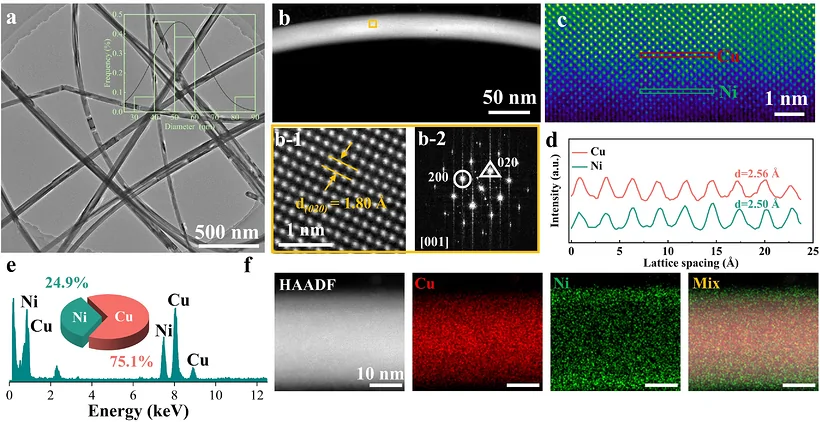
TEM characterization of the CuNi NWs. (a) Low‐Magnification TEM image of CuNi NWs (the insert shows diameter distribution histograms). (b) HAADF‐STEM image of a typical NW with a yellow box inset showing a fast Fourier transform of the interior. (c) Atomic‐scale HAADF‐STEM image of the heterointerface in CuNi NWs. (d) Intensity profiles of the red and green regions marked in (c). (e, f) STEM‐EDS spectra and elemental mapping.

As the central subject of this study, the in situ reconstructed CuNi NT was thoroughly characterized by STEM and EELS. The NT exhibits a ∼8 nm‐thick wall and an ultrathin surface layer (2–3 nm), as confirmed by aberration‐corrected‐HAADF imaging (Figure 3a and Figure S12). Figure S13 exhibits expanded lattice spacings corresponding to the $\left(\bar{1}1\bar{1}\right)$ and (200) planes of Ni (Region 1), suggesting partial Cu retention within the Ni lattice after electrochemical etching. This Cu–Ni alloying is further supported by EDS elemental mapping (Figure S14). Local magnification of region 2 in the CuNi NT demonstrates atomic‐scale rearrangement and high‐index surface steps induced by potential cycling (Figure 3b). FFT patterns of Figure 3c reveal the *fcc* crystalline NT wall with an amorphous surface shell in region 3, featuring ordered‐disordered heterointerfaces (Figure 3d), a structural motif that is favorable for enhanced electrocatalytic performance. The measured crystalline *d*‐spacings in Figure 3d can be assigned to the alloy NT wall. The NTs are further characterized from a top‐view perspective. In Figure 3e, the cross‐sectional results well evidence the hollow feature and crystalline–amorphous heterostructure. Despite potential‐driven Cu dissolution and migration, the *fcc* five‐fold symmetry architecture remains preserved, demonstrating excellent structural inheritance from the CuNi NW. Such twinning has been reported to confer good conductivity and mechanical robustness [37, 38], which could enhance structural and property continuity under harsh reaction conditions.

**FIGURE 3:**
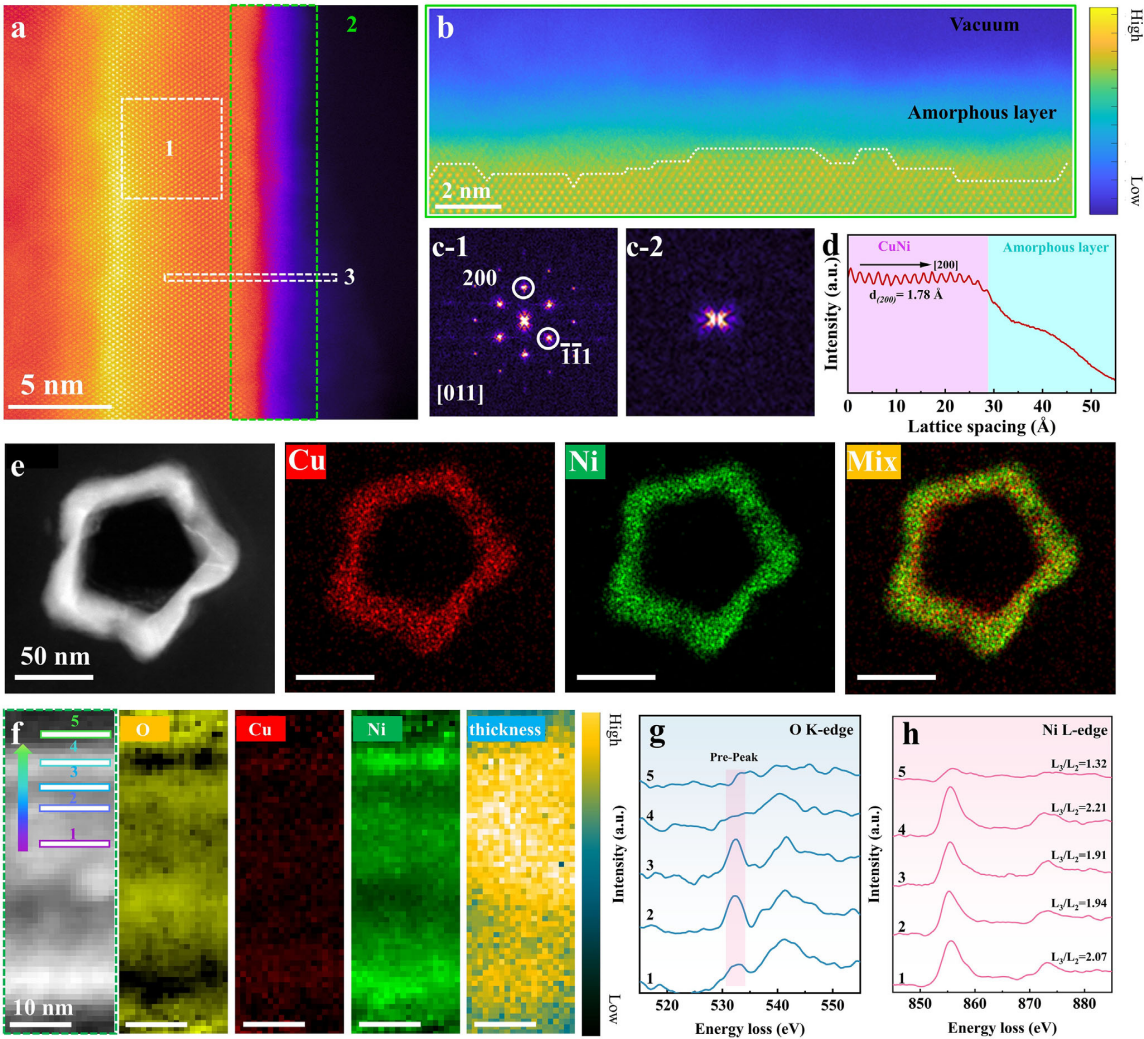
TEM characterization of the CuNi NTs. (a) Atomic‐scale HAADF‐STEM image of a typical NT with three regions marked. (b, c) Atomic‐scale HAADF‐STEM image of the heterointerface, i.e., region 2 in (a), with a fast Fourier transform of the crystalline–amorphous regions in (c). (d) Intensity profiles of region 3 in (a). (e) EDS elemental mapping image from an in‐top view. (f) EELS elemental mapping of the CuNi NTs. (g) O K‐edge EELS spectra. (h) Ni L‐edge EELS spectra.

To elucidate the electronic structure evolution associated with crystallography reconstruction, EELS has been conducted to investigate the localized valence state. Line‐scan EELS in Figure 3f reveals distinct O K‐edge and Ni L‐edge signatures across the crystalline–amorphous interface (Figure 3g,h). Spectral imaging further resolves the spatial distribution of O, Ni, and Cu, and quantifies the O: Ni atomic ratio along the scan path (Figure S15). Notably, elevated O: Ni ratios are observed in both hollow interiors and tube‐wall interfaces, indicating enhanced Ni oxidation following reconstruction. Stronger O K‐edge pre‐peaks and reduced Ni L_3_/L_2_ white‐line ratios in regions 2 and 3 (vs. 1 and 4) suggest a decrease in oxygen vacancies and a higher Ni valence state (Figure 3g,h) [39]. Compared to pristine CuNi NWs, the CuNi NTs exhibit attenuated Ni white‐line intensities, further validating valence‐state modulation. XPS analysis provides complementary insights into the surface chemical states and adsorption characteristics (Figure S16). The XPS profiles exhibit presence of Ni^2+^ and Cu^2+^ species, with absence of discernible metallic (zerovalent) peaks. Notably, the Ni 2p spectra display dominant contributions from oxidized Ni^2+^ accompanied by characteristic satellite features, consistent with the formation of stabilized amorphous layer by electrooxidation. Deconvolution of O 1s spectra demonstrates a remarkable increase in the hydroxyl group (OH^−^) component (531.2 eV) compared to initial CuNi NWs (Table S2). This interfacial OH^−^ enrichment correlates strongly with the EELS‐identified oxygen‐rich domains, collectively confirming the formation of amorphous oxide layer during reconstruction. Above multiscale characterization collectively demonstrate that the potential‐driven reconstruction enables electrochemical self‐regulation of both atomic configuration and electronic states. The resulting crystalline–amorphous heterostructure is expected to offer operando adaptability, dynamically balancing structural stability with catalytic activation under OER conditions.

Figure 4 systematically compares the OER performances of the CuNi NT, NW, and reference samples in 1 M KOH. All measurements were conducted using a standard three‐electrode system, with potentials calibrated against the reversible hydrogen electrode (RHE). A series of CuNi NWs with different Cu:Ni ratios were prepared and tested. The sample with a Cu:Ni ratio of 3:1 exhibited the best OER performance and was thus chosen as the main research subject for further investigations (Figure S17). Initial OER activity evaluation via linear sweep voltammetry (LSV) measurement revealed that pristine CuNi NWs exhibited moderate activity, outperforming commercial IrO_2_ and Cu NWs but remaining suboptimal in comparison to CuNi NTs, suggesting the self‐optimization effect of electrochemical reconstruction (Figure 4a). CV activation led to OER performance stabilization after 2000 cycles (Figure S18). In addition, enhanced anodic peaks at ∼1.4 V vs. RHE correlated with Ni^2+^/Ni^3+^ redox processes and active NiOOH formation [40]. CuNi NTs demonstrate significantly enhanced OER kinetics, evidenced by a low Tafel slope of 69 mV dec^−1^ (Figure 4b,c), outperforming Cu NWs (159 mV dec^−1^), IrO_2_ (112 mV dec^−1^), and CuNi NWs (91 mV dec^−1^). Electrochemical impedance spectroscopy (EIS) fitted with the equivalent circuit reveals a markedly reduced charge transfer resistance (R_ct_) for CuNi NTs (Figure 4d and Table S3). Furthermore, double‐layer capacitance (C_dl_) was used to further evaluate the electrochemical active surface area (ECSA) of all samples (Figure 4e and Figures S19 and S20). CuNi NTs display the highest C_dl_ value (12.53 mF cm^−2^), exceeding those of CuNi NWs (5.73 mF cm^−2^) and IrO_2_ (1.62 mF cm^−2^), indicating a larger accessible surface and more abundant active sites, which is promoted by the hollow tubular architecture. Specific activity, normalized by ECSA, is also highest for CuNi NTs (323.4 µA cm^−2^ at η = 300 mV), outperforming CuNi NWs (55.6 µA cm^−2^), as shown in Figure 4f. To further evaluate the intrinsic activity of Ni species, we calculated the turnover frequency (TOF) at η = 300 mV. The results show that CuNi NTs deliver a TOF of 0.0052 s^−1^, which is higher than that of CuNi NWs (0.0013 s^−1^) (Figure S21). These results indicate that the prepared CuNi NTs possess excellent intrinsic activity. A comprehensive comparison of key performance metrics (Figure 4g) highlights the outstanding OER activity of CuNi NTs. Moreover, the CuNi NTs exhibit OER activity that is highly competitive with state‐of‐the‐art Ni‐based metals and oxides (Figure S22 and Table S4). The comprehensive comparison reveals that the NTs outperform both monometallic Ni sites and synergistic Ni–M dual‐site systems, validating the efficacy of our design paradigm.

**FIGURE 4:**
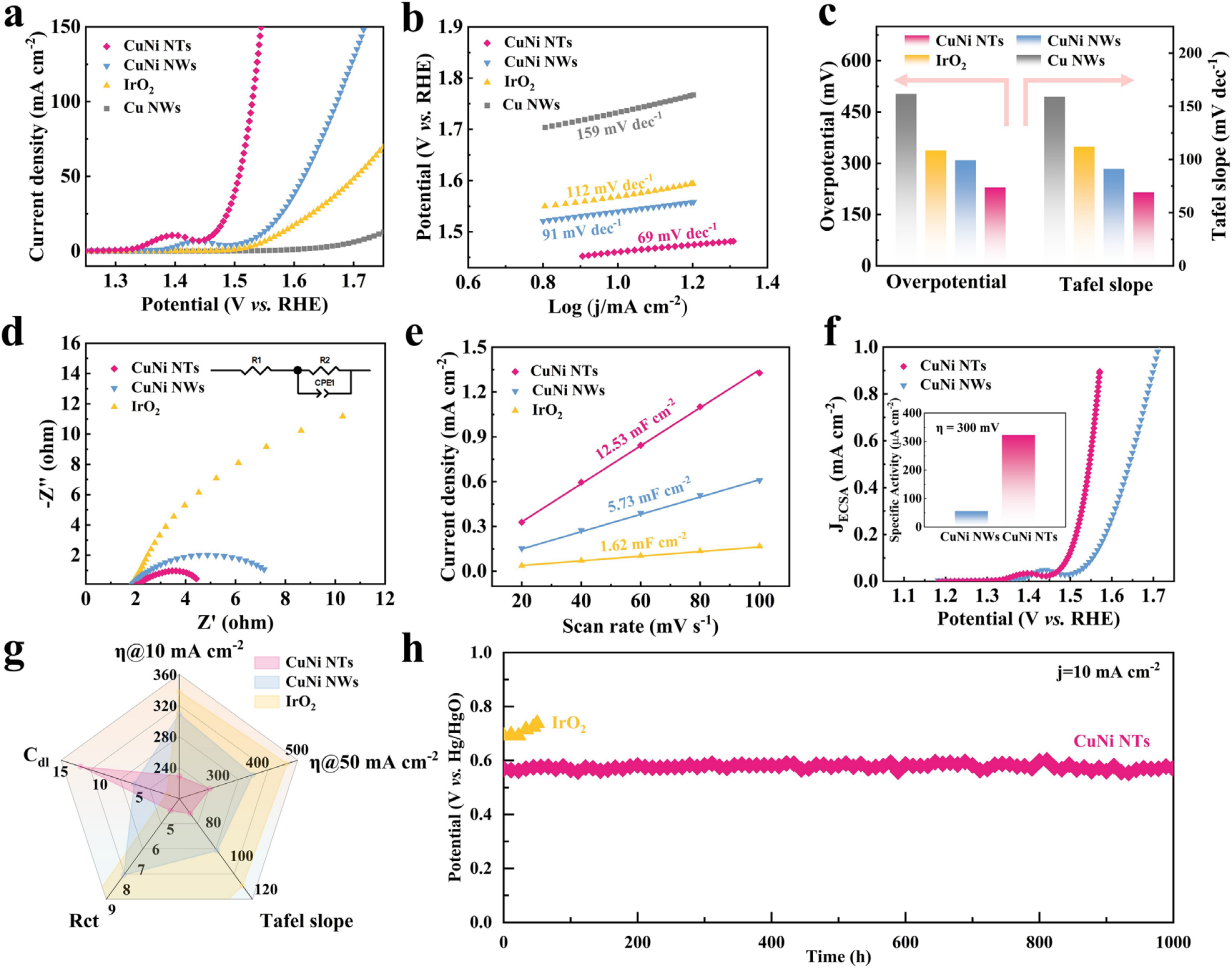
Electrochemical evaluation of catalysts. (a) Polarization curves of CuNi NTs, CuNi NWs, IrO_2_, and Cu NWs collected at a scan rate of 5 mV s^−1^. (b, c) Corresponding Tafel plots and overpotentials of various catalysts at 10 mA cm^−2^. (d) Electrochemical impedance spectroscopy (EIS) of CuNi NTs and reference electrocatalysts. (e) C_dl_ of CuNi NTs, CuNi NWs, and IrO_2_. (f) Polarization curves normalized by ECSA. (g) Schematic diagram for the summarization of OER performance. (h) Chronopotentiometry test of CuNi NTs and IrO_2_ at 10 mA cm^−2^.

As shown in Figure 4h, the catalyst stability has been assessed by chronopotentiometry at 10 mA cm^−2^, where CuNi NTs exhibit negligible degradation (only a 10 mV overpotential increase) after 1000 h, significantly outperforming the benchmark IrO_2_. TEM characterization on post‐stability test samples confirms the structural robustness of CuNi NTs, with the hollow configuration well preserved (Figure S23). EDS line scanning results further confirm that the CuNi NTs consist of a CuNi alloy core and a Ni‐rich shell (Figure S24). The combined analyses of XRD and XPS demonstrate that both the crystalline phase and the surface chemical states show negligible changes after the 1000 h stability test (Figures S25 and S26), verifying its excellent structural and chemical stability. Importantly, the amorphous reconstruction layer remains intact on the surface, effectively protecting the metallic component from dissolution during electrocatalytic cycles, which is highly desired in noble‐metal‐free catalysts [41, 42].

To gain mechanistic insights into the OER process, operando EIS and Raman spectroscopy have been employed to track the evolution of charge‐transfer kinetics and surface bonding configurations in CuNi NTs and NWs during catalysis. The EIS measurements were first conducted to elucidate the underlying mechanisms for the enhanced OER kinetics during the atomic knockout process. As shown in Figure S27, the Nyquist plots at different potentials reveal that the CuNi NTs exhibit the smallest semicircle, indicating superior electronic conductivity and faster charge transfer capability. Notably, the semicircle of the CuNi NTs decays more rapidly with increasing applied potential, indicating optimized polarizability of the CuNi NTs [43, 44]. In the Bode plots (Figure 5a,b), two distinct peaks emerge in different frequency regions: the high‐frequency peak (10^2^–10^3^ Hz), related to C_dl_ at the electrode–electrolyte interface, and the low‐frequency peak (10^0^–10^1^ Hz), attributed to the OER process [45]. Remarkably, the CuNi NTs demonstrate a larger capacitive response in the high‐frequency region under identical potentials, indicating stronger OH^−^ affinity. The OH^−^ accumulation‐induced C_dl_ enhancement contributes to an elevated overall catalytic driving force [46]. As shown in Figure 5c, a distinct phase angle emerges in the low‐frequency region when the potential reaches 1.4 V vs. RHE. Compared to the CuNi NWs, the CuNi NTs exhibit a smaller phase angle, signifying faster OER kinetics [47]. These results collectively demonstrate that the crystalline‐amorphous heterostructure facilitates OH^−^ adsorption and accelerates OER reaction dynamics.

**FIGURE 5:**
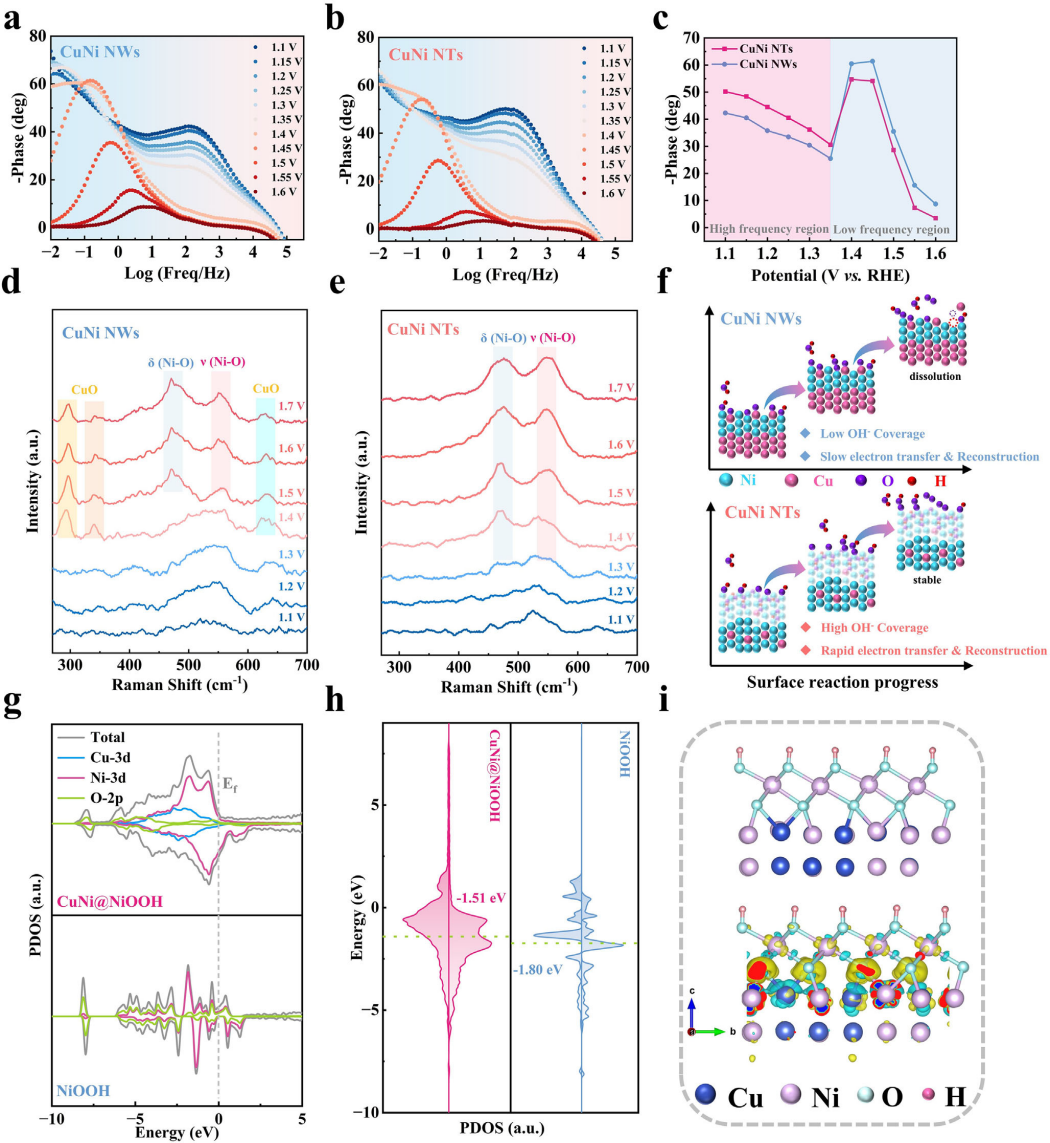
Structural and OER electrocatalytic mechanistic understandings of CuNi NWs and NTs. (a, b) Bode plots of CuNi NWs and CuNi NTs at a potential range from 1.1 to 1.6 V vs. RHE. (c) Phase angles measured at potentials between 1.1 V and 1.6 V vs. RHE. (d, e) Operando Raman spectra of CuNi NWs and CuNi NTs during the OER process. (f) Schematic comparison of the surface reaction for CuNi NTs and CuNi NWs. (g) PDOS of CuNi@NiOOH vs. NiOOH. (h) PDOS showing upward d‐band shift. (i) Charge redistribution at the CuNi@NiOOH interface.

The potential‐dependent Raman measurements were conducted in situ on both the CuNi NTs and NWs. As shown in Figure 5d, within the low‐potential range (1.1–1.3 V vs. RHE), a shallow shoulder peak centered at ∼500 cm^−1^ is observed in the CuNi NW spectrum, attributed to the surface‐oxidized Cu and Ni species, consistent with previous reports [48, 49]. When the potential increases to 1.4 V vs. RHE, three distinct characteristic peaks emerge at 296, 346, and 631 cm^−1^, corresponding to Raman peak of CuO, which could result from the oxidation of internal Cu atoms during the OER process [50]. Under harsh OER conditions, Cu atoms are selectively knocked out, and the faster diffusion of Cu^+^ relative to O^−^ drives the outward migration of the Cu/CuO interface, generating internal voids as confirmed by TEM [32]. Notably, two characteristic Raman peaks at 473 cm^−1^ and 554 cm^−1^, respectively, corresponding to the E_g_ (δ _Ni‐O_) and A_1g_ (ν _Ni‐O_) modes of NiOOH, can be identified in both CuNi NW and NT (Figure 5d,e). Relative to NWs, NTs require a reduced potential threshold to initiate NiOOH evolution. This facilitated in situ reconstruction establishes a mechanistic basis for the enhanced OER kinetics [51]. Another notable feature is the absence of CuO‐related vibrational peaks in the NTs, indicating the protective role of the in situ formed amorphous layer on suppressing core oxidation and ensuring structural integrity. This merit delivers significant stability for OER electrocatalysis. Figure 5f schematically compares the surface evolution and OER kinetics of the NWs and NTs under potentials, summarizing the advantageous high OH^−^ coverage and faster electron transfer of CuNi NTs.

Preliminary DFT calculations on the crystalline–amorphous interface have been carried out to elucidate the electronic origin of enhanced OER performance. As shown in Figure 5g, the projected density of states (PDOS) of CuNi@NiOOH displays a relative increase in DOS near the Fermi level, confirming its enhanced electrical conductivity and significantly accelerating OER kinetics. The interfacial coupling leads to an upward shift of the *d*‐band center (from −1.80 eV for NiOOH to −1.51 eV for CuNi@NiOOH), suggesting the favorable adsorption of key OER intermediates, as shown in Figure 5h. To further visualize the interfacial charge redistribution, the differential charge density maps in Figure 5i further reveal electron accumulation around O atoms in NiOOH and depletion around interfacial Cu atoms, suggesting strengthened Ni─O bonding and partial Cu oxidation that stabilizes NiOOH against over‐oxidation, in line with our experimental characterizations. Moreover, enhanced charge density overlap highlights strong orbital hybridization that optimizes intermediate adsorption energies and promotes rapid electron transfer.

Taken together with the structural, electronic, and operando characterizations, the superior OER activity and stability of CuNi NTs can be attributed to three key factors. Firstly, NiO_x_ layers act as effective precatalysts that transform into NiOOH under OER operation conditions, ensuring rapid generation of high‐valent Ni^2+^/Ni^3+^ sites [40, 52]. Secondly, the interface between NiO_x_ and the CuNi metallic core promotes electronic coupling, facilitating charge redistribution and optimizing the adsorption energies of reaction intermediates (*OH, *O, *OOH) [53]. Thirdly, the amorphous shell serves as a protective layer, mitigating corrosion, suppressing Cu leaching, and preserving structural integrity. Therefore, the synergy of the facile NiOOH formation, optimized interfacial electronic structure, and structural protection accounts for the superior OER performance of CuNi NTs.

## 3 Conclusions

In conclusion, we have developed an electrochemical reconstruction strategy to engineer crystalline–amorphous CuNi NTs as noble‐metal‐free catalysts for OER. The CuNi NTs achieve 10 mA cm^−2^ at an overpotential of 229 mV and operate over 1000 h in alkaline conditions. Atomic‐resolution STEM and EELS, combined with in situ spectroscopies, reveal the structural and electronic signatures at the crystalline–amorphous interface and the dynamic reconstruction process. Preliminary DFT calculations reveal that the interfacial coupling in CuNi@NiOOH shifts the *d*‐band center and redistributes charge, enhancing intermediate adsorption and OER kinetics. The synergy between the reconstructed surface active sites and the hetero‐interface accelerates reaction kinetics, while the amorphous layer passivates the metallic core against oxidative degradation, effectively unifying catalytic activity with structural integrity. These findings provide a robust blueprint for engineering earth‐abundant catalysts that unite high activity with operational stability.

## Author Contributions

**Tian‐Yu Xia**: formal analysis. **Hai‐Zhong Guo**: formal analysi. **Ling‐Rui Wang**: formal analysis. **Han Gao**: conceptualization, data curation, supervision, resources, project administration, formal analysis, validation, investigation, funding acquisition, writing – review and editing. **Zhi‐Gang Chen**: conceptualization, resources, project administration, formal analysis, funding acquisition, investigation, writing – review and editing. **Shi‐Yu Zhu**: methodology, software, visualization, writing – original draft. **Xiao‐Lei Shi**: writing – review and editing. **Meng Li**: software. **Wei‐Di Liu**: formal analysis.

## Conflicts of Interest

The authors declare no conflicts of interest.

## Supporting information




**Supporting File**: advs76436‐sup‐0001‐SuppMat.docx.

## Data Availability

The data that support the findings of this study are available from the corresponding author upon reasonable request.
