# Supplementary material for: In Situ Reconstructed Crystalline–Amorphous CuNi Nanotubes Unifying Activity and Stability for Oxygen Evolution
Source: Adv Sci (Weinh). 2026 Jul 6;13(54):e76436. doi: 10.1002/advs.76436 (PMC13335110; doi:10.1002/advs.76436)
Supplement: Supplementary file 1 [file ADVS-13-e76436-s001.docx]

**Supporting Information**

***In situ* Reconstructed Crystalline–Amorphous CuNi Nanotubes Unifying Activity and Stability for Oxygen Evolution**

Shi-Yu Zhu^1,†^, Han Gao^1,2,†,^*, Meng Li^2^, Ling-Rui Wang^1^, Tian-Yu Xia^1^, Wei-Di Liu^2^, Xiao-Lei Shi^2^, Hai-Zhong Guo^1,3,^*, Zhi-Gang Chen^2,^*

^†^indicates authors are equally contributing: Shi-Yu Zhu, Han Gao

^*^indicates the corresponding authors: Han Gao, Hai-Zhong Guo, Zhi-Gang Chen

**Affiliations**

^1^ *School of Physics, Zhengzhou University, Zhengzhou, 450001, China*

^2^ *School of Chemistry and Physics, ARC Research Hub in Zero-emission Power Generation for Carbon Neutrality, and Centre for Materials Science, Queensland University of Technology, Brisbane, Queensland, Australia*

^3^ *Institute of Quantum Materials and Physics, Henan Academy of Sciences, 450046, Zhengzhou, China*

Han Gao: [hgao@zzu.edu.cn](mailto:hgao@zzu.edu.cn)

Hai-Zhong Guo: [hguo@zzu.edu.cn](mailto:hguo@zzu.edu.cn)

Zhi-Gang Chen: [zhigang.chen@qut.edu.au](mailto:zhigang.chen@qut.edu.au)

**1. Experimental Section.**

**1.1 Chemicals and reagents.**

Copper (II) acetylacetonate (Cu(acac)_2_) and Nickel (II) acetylacetonate (Ni(acac)_2_) were purchased from Macklin. Iron (III) chloride hexahydrate (FeCl_3_·6H_2_O), Iridium oxide (IrO_2_), and Oleylamine (OAm) were purchased from Aladdin. Ascorbic acid (AA) was purchased from Xilong Scientific. All the materials were used without further treatment.

**1.2 Synthesis of the One-dimensional CuNi nanowires (CuNi NWs).**

For the synthesis of CuNi nanowires, Cu(acac)_2_ (20 mg), Ni(acac)_2_ (20 mg), FeCl_3_·6H_2_O (28 mg), ascorbic acid (105.6 mg), and 10 mL of OAm were introduced into a 30 mL glass vial. The mixture was ultrasonicated for 1 h to achieve a homogeneous dispersion, after which the homogeneous mixture was rapidly heated to 180 °C and maintained at this temperature for 6 h in an oil bath. Upon completion, the reaction mixture was allowed to cool to room temperature. The resulting product was collected by centrifugation and repeatedly washed with a hexane/ethanol mixed solvent.

By adjusting the amount of Ni(acac)_2_ precursor to 18 mg and 24 mg, we successfully fabricated Cu_80_Ni_20_ NWs and Cu_65_Ni_35_ NWs, respectively.

The synthesis of Cu Nanowires are similar, except that Ni(acac)_2_ (20 mg) was removed from the precursors, and the reaction temperature was adjusted from 180 °C (for 6 h) to 170 °C (for 3 h).

**1.3 Synthesis of the One-dimensional CuNi nanotubes (CuNi NTs).**

The as-obtained CuNi NWs were loaded onto carbon cloth, followed by cyclic voltammetry (CV) activation in 1 M KOH solution for 2,000 cycles. The activation was performed within a voltage range of 1.2—1.7 V vs RHE at a scan rate of 5 mV s⁻^1^.

**1.4 Characterizations.**

Transmission electron microscopy (TEM) images were obtained on JEOL JEM-2100 and Thermofisher (FEI) Talos F200s microscope with an accelerating voltage of 200 kV. High-resolution scanning transmission electron microscopy (HR-STEM) images and energy-dispersive X-ray spectroscopy (EDS) were acquired using a FEI Spectra 300 double Cs-corrected (S)TEM operating at an accelerating voltage of 300 kV. The crystal structure of the samples was determined by X-ray diffraction (XRD, PANalytical Empyrean), while X-ray photoelectron spectroscopy (XPS) measurements were performed using a Thermo Scientific K-Alpha system. Inductively coupled plasma mass spectrometry (ICP-MS, Agilent 7800) was employed to determine the chemical element types and their contents in the samples. *In situ* Raman spectroscopic measurements were carried out with a spectrometer (HRS-500MS, Princeton Instruments) equipped with 1,200 lines/mm gratings and a high-sensitivity thermoelectrically cooled CCD (PIX-256E, Princeton Instruments) using a 532 nm laser excitation source.

**1.5 Electrochemical measurements.**

All electrochemical measurements were conducted at room temperature using a CH Instruments 660E workstation (Shanghai Chenhua) equipped with a three-electrode configuration. For OER measurements, the catalysts supported on carbon cloth (active area 1 cm×1 cm) served as the working electrode, while a Hg/HgO electrode and a carbon rod were employed as the reference and counter electrodes, respectively.

To prepare the catalyst ink, 4 mg of catalyst was ultrasonically dispersed in 2 mL deionized water and 2 mL ethanol, followed by the addition of 8 µL of 5.0 wt.% Nafion solution to obtain a homogeneous mixture. Subsequently, 2 mL of the ink was drop-cast onto a carbon cloth substrate and dried at room temperature (catalyst loading: 2 mg cm⁻^2^). All tests were performed in 1 M KOH solution. The potentials were converted to the reversible hydrogen electrode (RHE) scale according to the equation:

$E (vs RHE) = E (vs Hg/HgO) + 0.098 V + 0.0591 \times pH$	(1)

Linear sweep voltammetry (LSV) was performed between 1.1 and 1.8 V *vs* RHE at a scan rate of 5 mV s⁻^1^. LSV curves were corrected with 85% ohmic iR drop compensation.

The overpotential 𝜂 is calculated according to the following equation:

$\eta= E(RHE) - 1.23 V$	(2)

Tafel slopes were derived from the corresponding polarization data using the standard Tafel equation:

$\eta= a + blog(j)$	(3)

Electrochemical impedance spectroscopy (EIS) was carried out over a frequency range from 0.01 to 10^5^ Hz. The double layer capacitance (C_dl_) is measured by CV in the non-Faraday region from 0.15 to 0.25 V *vs* Hg/HgO at various scan rates. The electrochemical active surface area (ECSA) of catalyst was calculated by the equation:

$ECSA = C_{dl}/C_{s}$	(4)

Where C_s_ refers to the specific capacitance with a value of 40 μF cm⁻^2^.[1]

The TOF value was calculated in this work using the equation:

$TOF = \frac{j_{geo} \times A}{4\times e\times n}$	(5)

In this equation: $j_{geo}$ is the uncompensated current density at η = 300 mV, A is the geometric area of carbon cloth (1 cm^2^), e is the electron charge (1.602$\times$10⁻^19^ C), and n is the number of active sites per electron.

The number of active sites (n) was calculated based on the total loading mass of the catalyst. Herein, all Ni metal atoms were assumed to serve as active sites, and the calculation was performed using the following formula:

$n_{mass} = \frac{m_{loading} \times N_{A}}{M_{w}}\times n_{Ni}$	(6)

In this equation: $m_{loading}$ is the loading mass; N_A_ is Avogadro's constant (6.022 $\times$ 10^23^ mol⁻^1^); Mw is the molecular weight of catalysts and n_Ni_ is the number of Ni atom per molar of catalysts.

**1.6 DFT Calculations.**

Density functional theory (DFT) calculations were performed using the Vienna Ab-initio Simulation Package (VASP).[2] The exchange-correlation interactions were described within the generalized gradient approximation (GGA) using the Perdew-Burke-Ernzerhof (PBE) functional.[3-4] A cutoff energy of 550 eV was employed for structural optimizations. A 15 Å vacuum slab was employed along the z-direction. A Monkhorst-Pack k-point mesh of 5 × 3 × 1 was utilized in the calculations. All structures were fully optimized until the total energy converged to 1 × 10⁻^5^ eV and the residual force on each atom converged to 0.05 eV Å⁻^1^. To consider the van der Waals interactions, the DFT-D3 method was applied.[5]

**2. Supplementary Figures and Tables.**

**Figure S1.** (a) HAADF-STEM image of CuNi NTs combined with elemental distribution maps of Cu and Ni after 500 cycles. (b) Atomic fraction of Cu and Ni in the CuNi NTs.

**Figure S2.** (a) HAADF-STEM image of CuNi NTs after 1,900 cycles. (b, c) EDS mapping of CuNi NTs. (d) Atomic fraction of Cu and Ni in the CuNi NTs.

**Figure S3.** (a) HAADF-STEM image of CuNi NTs after 2,100 cycles. (b, c) EDS mapping of CuNi NTs. (d) Atomic fraction of Cu and Ni in the CuNi NTs.

**Figure S4.** (a) HAADF-STEM image of CuNi NTs after 2,300 cycles. (b, c) EDS mapping of CuNi NTs. (d) Atomic fraction of Cu and Ni in the CuNi NTs.
**Figure S5.** Atomic fraction of Cu and Ni as a function of cycle number.

**Figure S6.** GPA strain map of CuNi NWs corresponds to Figure 2c. (a) Mean dilatation. (b-d) correspond to the ε_xy_, ε_xx_, and ε_yy_ directions, respectively.

**Figure S7.** EDS line-scan elemental profiles of CuNi NWs.

**Figure S8.** (a) STEM-EDS elemental mapping from the top view. (b) HAADF-STEM image. (c) Atomic-scale HAADF-STEM image of the NWs (corresponding to yellow box) with a fast Fourier transform (d).

**Figure S9.** XRD spectra of CuNi NWs.

**Figure S10.** (a) EELS spectra of the CuNi NWs were recorded along the colored arrows. (b) O K-edge EELS spectra. (c) Ni L-edge EELS spectra. (d) STEM-EELS mapping distribution of Cu, Ni, and O in the CuNi NWs. (e) Cu L-edge EELS spectra.

**Figure S11.** XPS spectra of CuNi NWs. (a) Survey spectrum. (b-d) High-resolution XPS spectra of Cu 2p, Ni 2p, and O 1s.

**Figure S12.** (a) Low-magnification HAADF-STEM image. (b) Heterointerface characterization of CuNi NTs. (c) 3D atom-overlapping Gaussian function fitting result for the yellow dashed box in (b).

**Figure S13.** (a) Atomic-scale HAADF image of the marked area 1 in Figure 3. (b, c) Intensity profile along [$\bar{1}$1$\bar{1}$] and [200], respectively.

**Figure S14.** (a) STEM-EDS elemental mapping. (b) Intensity profiles of the marked in (a). (c) Atomic fraction of Cu, Ni, and O.

**Figure S15.** Ni/O atomic fraction of CuNi NTs measured by EELS, recorded from 1 to 5 in Figure 3f.

**Figure S16.** XPS spectra of CuNi NTs. (a-c) High-resolution XPS spectra of Cu 2p, Ni 2p, and O 1s.

**Figure S17.** (a) Polarization curves of CuNi NWs with different Cu/Ni ratios were collected at a scan rate of 5 mV s⁻^1^. (b) Corresponding overpotential of various catalysts at 10 mA cm^−2^.

**Figure S18.** (a) Polarization curves as the function of cycle number. (b) Corresponding overpotential variation.

**Figure S19.** CV curves of (a) CuNi NTs, (b) CuNi NWs and (c) IrO_2_ at different scan rates.

**Figure S20.** ECSA of CuNi NWs and CuNi NTs.

**Figure S21.** TOF of CuNi NWs and CuNi NTs.

**Figure S22.** The OER activity (overpotential at 10 mA cm^−2^) comparison between CuNi NTs and recently reported representative Ni-based OER electrocatalysts.

**Figure S23.** (a) Low-magnification TEM image of CuNi NTs. (b) EDS elemental mappings of Ni and Cu after Chronopotentiometry test. (c) Cu and Ni atomic fraction.

**Figure S24.** (a) HAADF-STEM image of CuNi NTs after 1,000 h stability test. (b-d) EDS mapping of single CuNi NT. (e) Line-scan EDS profile of the corresponding single CuNi NT.

**Figure S25.** XRD spectra of CuNi NTs and after 1,000 h stability test.

**Figure S26.** High-resolution XPS spectra of (a) Cu 2p and (b) Ni 2p for CuNi NTs before and after OER.
**Figure S27.** Nyquist plots for (a) CuNi NWs and (b) CuNi NTs at different applied potentials versus RHE in 1 M KOH.

**Table S1.** ICP-MS results for CuNi NWs and CuNi NTs.

| **Sample** | **Cu (at. %)** | **Ni (at. %)** |
| --- | --- | --- |
| **CuNi NWs** | **75** | **25** |
| **CuNi NTs** | **33** | **67** |
| **CuNi NTs after 1,000 h stability test** | **24** | **76** |

**Table S2.** XPS fitting analysis for the O 1s in CuNi NWs and CuNi NTs.

| **Sample** | **Peak** | **Proportion (%)** |
| --- | --- | --- |
| **CuNi NWs** | M-O | 28.98 |
|  | M-OH | 59.03 |
|  | Ad H_2_O | 11.99 |
| **CuNi NTs** | M-OH | 67.12 |
|  | Ad H_2_O | 28.27 |
|  | O in sulfonic acid group in Nafion | 4.61 |

**Table S3.** Fitted element parameters of the EIS equivalent circuit for CuNi NTs and CuNi NWs.

| Element | R1 | R2 | CPE1-T | CPE1-P |
| --- | --- | --- | --- | --- |
| CuNi NWs | 1.87 | 6.12 | 0.0074794 | 0.75238 |
| CuNi NTs | 2.176 | 2.72 | 0.7984 | 0.807664 |

**Table S4.** **Comparison of the overpotential between our CuNi NTs and recently reported Ni-based OER electrocatalysts in alkaline media**.

| Catalyst | Electrolyte | Overpotential@10 mA cm^-2^ | Ref |
| --- | --- | --- | --- |
| ***CuNi NTs*** | ***1M KOH*** | ***229*** | ***This work*** |
| Ni-TDC_0.6_BDC_0.4_ | 1M KOH | 230 | [6] |
| Cu_3.8_Ni@C | 1M KOH | 233 | [7] |
| CuNi@NiSe | 1M KOH | 293 | [8] |
| CuNi/FTO | 1M KOH | 294 | [9] |
| CeNi_3_-H/CC | 1M KOH | 296 | [10] |
| Ni_59_Cu_19_P_9_ | 1M KOH | 307 | [11] |
| Cu_50_Ni_50_ NP | 1M KOH | 318 | [12] |
| NiFeMn@triMPi | 1M KOH | 233 | [13] |
| NiCuFeP-20 | 1M KOH | 236 | [14] |
| Ni_58_Fe_17_Mo_25_ | 1M KOH | 239 | [15] |
| a/c-NiFe-G | 1M KOH | 250 | [16] |
| Ce-NiCo_2_O_4_ | 1M KOH | 270 | [17] |
| Fe-NiO/NiS_2_ | 1M KOH | 270 | [18] |
| Ni-Co alloy | 1M KOH | 288 | [19] |
| NiO_x_/NiFeO_x_-200 | 1M KOH | 289 | [20] |
| NiCo_2_O_4_-F_1_ | 1M KOH | 300 | [21] |
| Ni_0.8_Co_0.12_Mo_0.08_O | 1M KOH | 336 | [22] |

**References**

1. C. C. L. McCrory, S. Jung, I. M. Ferrer, S. M. Chatman, J. C. Peters and T. F. Jaramillo, “Benchmarking Hydrogen Evolving Reaction and Oxygen Evolving Reaction Electrocatalysts for Solar Water Splitting Devices,” *Journal of the American Chemical Society* 137 (2015): 4347-4357, <http://doi.org/10.1021/ja510442p>.

2. G. Kresse and J. Furthmüller, “Efficient iterative schemes for ab initio total-energy calculations using a plane-wave basis set,” *Physical Review B* 54 (1996): 11169-11186, <http://doi.org/10.1103/PhysRevB.54.11169>.

3. B. Hammer, L. B. Hansen and J. K. Nørskov, “Improved adsorption energetics within density-functional theory using revised Perdew-Burke-Ernzerhof functionals,” *Physical Review B* 59 (1999): 7413-7421, <http://doi.org/10.1103/PhysRevB.59.7413>.

4. J. P. Perdew, K. Burke and M. Ernzerhof, “Generalized Gradient Approximation Made Simple,” *Physical Review Letters* 77 (1996): 3865-3868, <http://doi.org/10.1103/PhysRevLett.77.3865>.

5. S. Grimme, J. Antony, S. Ehrlich and H. Krieg, “A consistent and accurate ab initio parametrization of density functional dispersion correction (DFT-D) for the 94 elements H-Pu,” *The Journal of Chemical Physics* 132 (2010): 154104, <http://doi.org/10.1063/1.3382344>.

6. Z. Huang, B. Wu, S. Zhang, et al., “Spin-State Engineering of Ni Centers by Dual-Ligand Competitive Coordination for Superior Oxygen Evolution Reaction,” *Angewandte Chemie International Edition* n/a (2026): e9878440, <https://doi.org/10.1002/ange.9878440>.

7. M. Kumar, D. I. Jeong and D. H. Yoon, “Copper nickel alloy nanorods textured nanoparticles for oxygen evolution reaction,” *Electrochimica Acta* 333 (2020): 135545, <https://doi.org/10.1016/j.electacta.2019.135545>.

8. D. Cao, J. Shao, Y. Cui, L. Zhang and D. Cheng, “Interfacial Engineering of Copper–Nickel Selenide Nanodendrites for Enhanced Overall Water Splitting in Alkali Condition,” *Small* 19 (2023): 2301613, <https://doi.org/10.1002/smll.202301613>.

9. S. Manzoor, T. Munawar, S. Gouadria, et al., “Nanopetals shaped CuNi alloy with defects abundant active surface for efficient electrocatalytic oxygen evolution reaction and high performance supercapacitor applications,” *Journal of Energy Storage* 55 (2022): 105488, <https://doi.org/10.1016/j.est.2022.105488>.

10. Z. Chen, S.Yao, J. Xu, et al., “Hydrogen-Induced Amorphization of Superlattice Cerium Nickel Intermetallics Enabling Efficient Alkaline Oxygen Evolution Reaction,” *Advanced Science* 13 (2026): e74479, <https://doi.org/10.1002/advs.74479>.

11. B. K. Kim, S.-K. Kim, S. K. Cho and J. J. Kim, “Enhanced catalytic activity of electrodeposited Ni-Cu-P toward oxygen evolution reaction,” *Applied Catalysis B: Environmental and Energy* 237 (2018): 409-415, <https://doi.org/10.1016/j.apcatb.2018.05.082>.

12. E. Gioria, S. Li, A. Mazheika, R. Naumann d'Alnoncourt, A. Thomas and F. Rosowski, “CuNi Nanoalloys with Tunable Composition and Oxygen Defects for the Enhancement of the Oxygen Evolution Reaction**,” *Angewandte Chemie International Edition* 62 (2023): e202217888, <https://doi.org/10.1002/anie.202217888>.

13. X. Jiang, Q. Yu, Z. Wen, et al., “Structural Engineering of Crystalline–Amorphous NiFeMn Alloy and Trimetallic Phosphite Heterostructure Electrocatalysts for High-Performance Industrial Anion Exchange Membrane Water Electrolysis,” *ACS Applied Materials & Interfaces* 17 (2025): 63296-63307, <http://doi.org/10.1021/acsami.5c11464>.

14. P. Cui, K. Peng, F. Miao and T. Gu, “Activating the Oxygen Evolution Performance of NiCuFe by Phosphorus Doping,” *Langmuir* 41 (2025): 25306-25314, <http://doi.org/10.1021/acs.langmuir.5c02648>.

15. Y. Jia, X. Ma, Y.-B. Yang, et al., “Synthesis, Alloying Process, and Enhanced Oxygen Evolution Activity of Highly Stable Ni–Fe–Mo Nanoparticles with a Face-Centered Cubic Phase,” *Small* 21 (2025): e05716, <https://doi.org/10.1002/smll.202505716>.

16. Z. Gong, R. Liu, H. Gong, et al., “Constructing a Graphene-Encapsulated Amorphous/Crystalline Heterophase NiFe Alloy by Microwave Thermal Shock for Boosting the Oxygen Evolution Reaction,” *ACS Catalysis* 11 (2021): 12284-12292, <http://doi.org/10.1021/acscatal.1c03333>.

17. X. Wang, J. Hu, T. Lu, et al., “Importing Atomic Rare-Earth Sites to Activate Lattice Oxygen of Spinel Oxides for Electrocatalytic Oxygen Evolution,” *Angewandte Chemie International Edition* 64 (2025): e202415306, <https://doi.org/10.1002/anie.202415306>.

18. N. Zhang, Y. Hu, L. An, et al., “Surface Activation and Ni-S Stabilization in NiO/NiS_2_ for Efficient Oxygen Evolution Reaction,” *Angewandte Chemie International Edition* 61 (2022): e202207217, <https://doi.org/10.1002/anie.202207217>.

19. F. Zhu, X. Wen, X. Li, et al., “Tuning the Wettability of Binary Alloy Electrodes to Promote OER Catalysis in Alkaline Media by Group Distribution Ratio,” *ACS Applied Energy Materials* 8 (2025): 1210-1219, <http://doi.org/10.1021/acsaem.4c02717>.

20. G. Shi, H. Osada, D. A. Tryk, et al., “Tuning the Ni-Oxide Layer Structure on NiFe Alloys for High Oxygen Evolution Reaction Activity,” *ACS Catalysis* 15 (2025): 14100-14107, <http://doi.org/10.1021/acscatal.5c02676>.

21. Y. Yue, X. Zhong, M. Sun, et al., “Fluorine Engineering Induces Phase Transformation in NiCo_2_O_4_ for Enhanced Active Motifs Formation in Oxygen Evolution Reaction,” *Advanced Materials* 37 (2025): 2418058, <https://doi.org/10.1002/adma.202418058>.

22. G. Shi, T. Tano, T. Iwataki, et al., “Highly Active Nanostructured NiCoMo-Based Catalyst for Oxygen Evolution in Anion-Exchange Membrane Water Electrolysis,” *ACS Applied Energy Materials* 6 (2023): 10742-10747, <http://doi.org/10.1021/acsaem.3c02152>.
